# An Assessment of Clinician Knowledge of Hidradenitis Suppurativa: Insights from a Multidisciplinary Survey Study

**DOI:** 10.3390/jcm14093171

**Published:** 2025-05-03

**Authors:** Klaudia Knecht-Gurwin, Adam Gurwin, Magdalena Łyko, Tomasz Drewa, Wojciech Kielan, Agnieszka Mastalerz-Migas, Rafał Stojko, Jacek C. Szepietowski, Lukasz Matusiak

**Affiliations:** 1 University Centre of General Dermatology and Oncodermatology, Wroclaw Medical University, 50-556 Wroclaw, Poland; lykomagdalena@gmail.com; 2 University Center of Excellence in Urology, Department of Minimally Invasive and Robotic Urology, Wroclaw Medical University, 50-556 Wroclaw, Poland; gurwin.adam@gmail.com; 3 General and Oncological Urology Clinic, Nicolaus Copernicus University in Toruń, 85-094 Bydgoszcz, Poland; t.drewa@wp.pl; 4 2nd Department of General Surgery and Surgical Oncology, Wroclaw Medical University, 50-556 Wroclaw, Poland; wojciech.kielan@umw.edu.pl; 5 Department of Family Medicine, Wroclaw Medical University, 51-141 Wroclaw, Poland; agnieszka.mastalerz-migas@umw.edu.pl; 6 Department of Gynecology, Obstetrics and Gynecologic Oncology, Medical University of Silesia, 40-211 Katowice, Poland; rstojko@sum.edu.pl; 7 Faculty of Medicine, Wroclaw University of Science and Technology, 50-377 Wroclaw, Poland; luke71@interia.pl; 8 Department of Dermato-Venereology, 4th Military Hospital, 50-981 Wroclaw, Poland

**Keywords:** hidradenitis suppurativa, skin diseases, clinical dermatology, dermatology education, diagnostic delay, misclassification, multidisciplinary survey, Hurley stage, medical education, physician awareness, early diagnosis, targeted interventions

## Abstract

**Background/Objectives:** Hidradenitis suppurativa (HS) is a chronic, debilitating skin disease primarily diagnosed through clinical examination. Despite its characteristic clinical features, HS remains under-recognized and frequently misclassified, especially by non-dermatologist clinicians. This study aims to evaluate the diagnostic accuracy of HS across various specialties, identify knowledge gaps, and inform targeted educational strategies to reduce diagnostic delays. **Methods:** A cross-sectional survey was conducted during multidisciplinary scientific conferences, enrolling 655 clinicians including dermatologists, gynecologists, urologists, general surgeons, and general practitioners. Participants were presented with clinical images representing HS lesions at Hurley stages I–III and responded to open-ended and closed-ended diagnostic questions. Data were analyzed to assess diagnostic accuracy and compare recognition patterns across specialties. **Results:** The recognition of HS varied significantly by specialty and disease stage. For Hurley stage III axillary disease, correct identification was highest among dermatologists (96.56%) compared to general practitioners (48.91%), gynecologists (31.25%), urologists (40%), and general surgeons (63.64%). In a Hurley II genital case in a male patient, only 34.5% diagnosed HS, while 25.65% suggested furunculosis and 16.18% venereal granuloma. For a Hurley I genital case in a female patient, 29.92% diagnosed HS, with furunculosis (23.36%) and steatocystoma multiplex (14.35%) as common misdiagnoses. A Hurley III buttock case was correctly identified by only 29.77% of participants. **Conclusions:** This large, first-of-its-kind global survey highlights substantial gaps in HS recognition, particularly among non-dermatologist clinicians. The findings underscore the urgent need for targeted, multidisciplinary educational interventions to improve diagnostic accuracy, reduce delays, and ultimately enhance patient outcomes in HS.

## 1. Introduction

Hidradenitis suppurativa (HS) is a chronic disorder afflicting apocrine gland regions of the skin. HS epitomizes a very debilitating ailment that instigates a significant depletion of quality of life [1]. Robust data on the worldwide prevalence of HS remain inconsistent [2,3,4]. The wide variations in prevalence may indicate that not all cases are correctly recognized or just possibly misclassified. Alongside the unfortunate translation of this disease entity in the ICD-10 classification where HS is listed as “acne inversa”—a term that may imply a subtype of acne rather than a distinct inflammatory disorder—patient hesitancy in seeking medical consultations due to the localization of lesions in intimate areas and a lack of awareness among physicians across various specialties appears to be a significant factor contributing to its inadequate recognition [5,6]. Approximately 7–10 years pass for the diagnosis to be established, and specifically, 65% of HS patients undergo at least five physician visits before receiving a diagnosis [7]. In the majority of cases, GPs are the first point of contact for HS patients. Despite subsequent consultations with dermatologists, GPs continue to serve as the main healthcare providers for 15% of patients who have received a diagnosis of HS. German data indicated that 30% of HS patients initially consult with a gynecologist; however, less than 5% of them receive a correct diagnosis [8].

This study aimed to confirm the assumed hypothesis, indicating that HS is insufficiently recognized and inaccurately classified among clinicians, and may pave the way for future initiatives aimed at raising awareness about this clinical entity. This would potentially lead to a reduction in the time taken to establish a diagnosis and alleviate the burden arising from the delayed implementation of treatment.

## 2. Materials and Methods

The cross-sectional survey (Appendix A) was conducted with the participation of dermatologists, gynecologists, urologists, general surgeons, and GPs. This study was conducted following approval from the research ethics committee (Reference 938/2021). Anonymous surveys were personally distributed at scientific congresses and conferences using QR codes, tablets, and paper-based questionnaires (WAWDERM 2023, the VI Warsaw Dermatological Days, 5–7 October 2023, Warsaw, Poland; The XI Conference on Controversies in Dermatology, 30 November–2 December 2023, Wrocław, Poland; The 53rd Scientific Congress of the Polish Urological Society 11–13 September 2023, Kraków, Poland; The Second Congress of Operative Gynecology 23–25 November 2023, Katowice, Poland; The 71st Congress of the Polish Surgeons’ Society, 20–23 September 2023, Wrocław, Poland; The XII Congress of the Polish Society of Family Medicine, 6–8 October 2023, Wrocław, Poland). Basic socio-demographic information regarding the respondents was collected. The surveys comprised 4 questions pertaining to socio-demographic aspects and 7 questions concerning the diagnosis and management of the patient. The questionnaires included images depicting skin lesions of HS at various stages according to the Hurley Score System—Hurley I, II, and III [9]. The first question was an open-ended inquiry soliciting the diagnosis of the disease, followed by subsequent closed-ended questions offering a selection among several proposed differential diagnoses. Additionally, a brief mention was included regarding the chronic and recurrent nature, with at least two exacerbations within 6 months, to meet the criteria outlined in Dessau 2006 for the diagnosis of HS [10]. Moreover, physicians were asked to suggest redirecting the patient to a specialist in another field, including GPs, dermatology, gynecology, urology, general surgery, gastroenterology, and infectious disease medicine. The set of images used was selected beforehand by two independent experts in HS (ŁM, JCSz) to ensure consensus and certainty regarding the diagnosis. Surveys that were not fully completed were excluded from the final analysis to ensure the accuracy of the data. Descriptive statistics for quantitative variables were presented as means with accompanying standard deviations (SD) and as medians with interquartile ranges (IQR), as appropriate. The normality of data distribution was assessed using the Shapiro–Wilk test. Categorical variables were analyzed using the chi-square (χ^2^) test or the Fisher–Freeman–Halton exact test, depending on data distribution and expected cell counts. The choice of test was determined by the structure of the contingency tables and sample size, with exact tests applied in cases of low expected frequencies. These analyses were used to compare diagnostic accuracy across specialties and career stages. A *p*-value < 0.05 was considered statistically significant. All analyses were performed using STATISTICA 13 software (TIBCO Software Inc., Palo Alto, CA, USA).

## 3. Results

A total of 750 participants were initially invited to complete the survey. Of these, 76 individuals declined participation, and 19 surveys were excluded from the analysis due to incomplete responses. Consequently, 655 fully completed surveys were included in the study, representing responses from 142 dermatologists, 154 general surgeons, 137 family medicine practitioners (GPs), 112 gynecologists, and 110 urologists. This yielded a response rate of 87.33%. The age of the respondents was 26–80 years (mean, 39 ± 10.55 years). Among the participants, 201 were in the midst of their specialization, 112 were specialists with less than 5 years since completing their specialization training, 50 were specialists with 5 to 10 years of experience post-specialization, and 138 were specialists with over 10 years since completing their specialization training.

**Figure 1:**
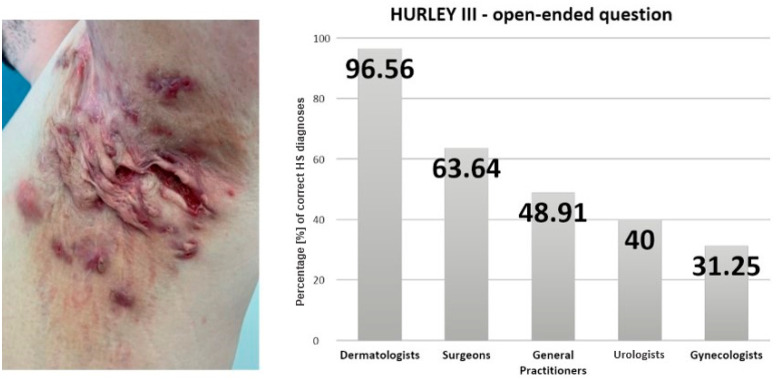
The charts with the percentage distribution of physician responses to open-ended questions identifying the condition as hidradenitis suppurativa, relative to their medical specialization.

In response to the initial open-ended question regarding the diagnosis of a male patient presenting with Hurley III stage disease localized in the axilla, the physicians provided the following responses: dermatologists diagnosed HS in 96.56% of cases, GPs in 48.91%, and gynecologists in 31.25% of cases. Among surveyed urologists, responses included HS in 40% of cases. General surgeons indicated a diagnosis of HS in 63.64% of cases. Statistical analysis confirmed a significant association between specialty and diagnostic accuracy (*p* < 0.001). These percentages are presented in Figure 1, which illustrates the distribution of correct responses by specialty. A detailed breakdown of all diagnostic responses, including alternative diagnoses selected by participants, is provided in Table 1.

Diagnostic accuracy also varied depending on the stage of specialization training. A detailed breakdown by specialty and career stage is presented in Table 2.

**Figure 2:**
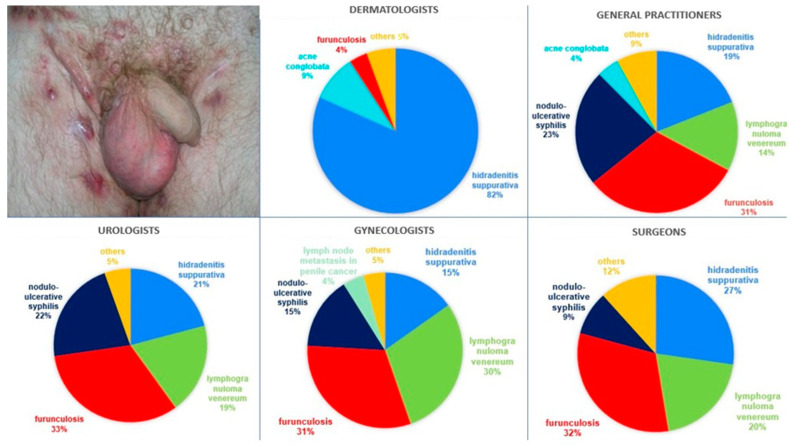
The male patient with Hurley II lesions in the perigenital area, alongside charts illustrating the percentage distribution of responses based on medical specialization. The results have been rounded for clarity of interpretation.

In response to the subsequent question regarding the genital area of male patient (Hurley II stage), the distribution of the most frequent responses was as follows: 34.5% of all surveyed physicians identified HS, 25.65% indicated furunculosis, and 16.18% venereal granuloma. Figure 2 illustrates the distribution of diagnoses across specialties. Delving into the specifics, dermatologists most frequently indicated the proper diagnosis, i.e., HS. Among physicians of other specialties, furunculosis was the most frequently chosen diagnosis, with 31.39% of GPs, 31.25% of gynecologists, 31.82% of general surgeons, and 32.73% of urologists providing such responses. Overall diagnostic accuracy of HS diagnosis differed significantly by specialty (*p* < 0.001). The discussed patient was most commonly referred to a dermatologist, with percentages of 81.02% among GPs, 66.07% among gynecologists, 66.88% among general surgeons, and 81.82% among urologists.

Detailed recognition rates by specialty and training stage are presented in Table 3.

**Figure 3:**
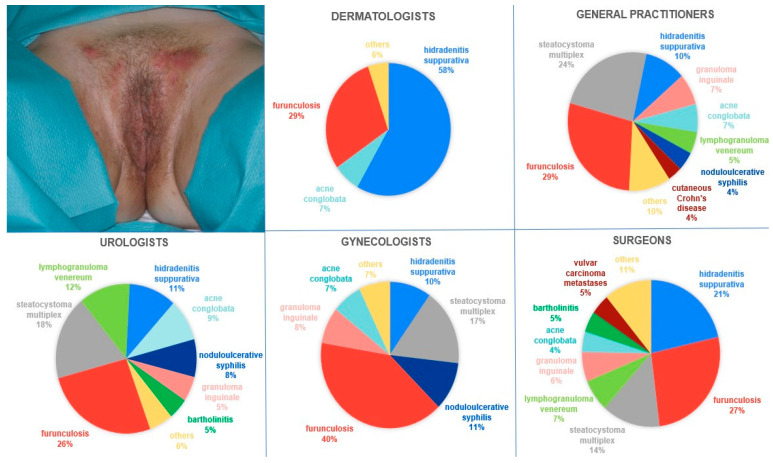
The female patient displaying lesions in the mons pubis and genital area classified as Hurley I stage, along with charts depicting the percentage distribution of proposed diagnoses. The results have been rounded for clarity of interpretation.

In the subsequent question featuring a photograph displaying the genital area of a female patient (with Hurley I stage), HS was most frequently identified by surveyed physicians (29.92%), furunculosis was identified in 23.36%, and steatocystoma multiplex was identified in 14.35%. Figure 3 presents the distribution of these responses across medical specialties. Among dermatologists, only 58.45% identified HS. Considering other medical specialties, furunculosis was most frequently diagnosed, with 28.57% of GPs, 40.18% of gynecologists, 26.62% of general surgeons, and 26.36% of urologists providing such responses. Statistical analysis confirmed significant variation in diagnostic patterns across medical specialties (*p* < 0.001). The patient was most frequently referred to a dermatologist, with 62.04% of GPs, 61.61% of gynecologists, 53.9% of general surgeons, and 72.73% of urologists providing such responses.

The relationship between diagnostic outcome and medical specialty was examined separately for each stage of specialization training. The aforementioned results are summarized in Table 4.

**Figure 4:**
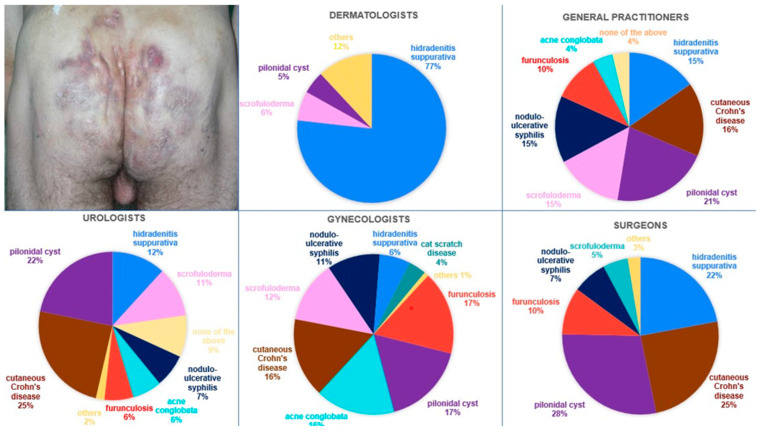
The male patient with lesions localized on the buttocks classified as Hurley III stage, accompanied by charts displaying the percentage distribution of proposed diagnoses. The results have been rounded for clarity of interpretation.

The last question pertained to a photograph illustrating the buttocks of a male patient displaying Hurley III stage. Figure 4 shows the distribution of responses by specialty. The most commonly indicated diagnoses were HS, pilonidal cyst, and the cutaneous manifestation of Crohn’s disease, accounting for 29.77%, 18.63%, and 16.79%, respectively. Among the surveyed physicians, 46.11%, 27.48%, and 13.89% would refer the patient to a dermatologist, general surgeon, and gastroenterologist, respectively. Scrutinizing the details, dermatologists accurately diagnosed HS in 76.76% of the cases. Within the surveyed GPs, the prevailing diagnosis was a pilonidal cyst (21.17%); 39.42% of GPs would refer the discussed patient to a dermatologist. In the cohort of surveyed gynecologists, the most frequent diagnoses were furunculosis (16.96%), and pilonidal cyst (16.96%); 46.43% of gynecologists would refer the discussed patient to a dermatologist. Amidst the general surgeons, the prevailing diagnosis was a pilonidal cyst (28.47%). Statistical analysis confirmed a significant association between specialty and diagnostic accuracy (*p* < 0.001). General surgeons indicated that this patient should be primarily managed by a general surgeon (44.16%). Urologists most commonly diagnosed cutaneous Crohn’s disease (24.55%). Overall, the discussed patient was most frequently referred to a dermatologist (40%).

The association between diagnostic accuracy and training stage was also evaluated with results subsumed in Table 5.

## 4. Discussion

The profound impact of HS on physical and emotional well-being underscores its significance beyond dermatological concerns, highlighting the need for interdisciplinary approaches in management. As clinicians dedicated to the holistic care of patients, it is imperative to confront the stark reality: the awareness of HS among multidisciplinary clinicians, including dermatologists, general surgeons, GPs, urologists, and gynecologists, remains alarmingly deficient.

To the best of our knowledge, this study represents the first study worldwide evaluating the ability of physicians across various specialties to identify HS based on clinical presentation. This focus is essential given that HS is a disorder primarily diagnosed based on its clinical manifestations.

Upon review of the literature, it is evident that patients diagnosed with HS often seek consultation from clinicians across various medical specialties, averaging approximately five physicians per patient encounter [7]. This trend underscores the considerable effort exerted by patients in their quest for diagnosis, despite the diagnostic process often enduring for up to a decade [8]. The etiology of this prolonged diagnostic journey remains incompletely elucidated. Presumably, clinicians who are not specialized dermatologists may lack sufficient familiarity with the condition. Nevertheless, it is noteworthy that more than 60% of surveyed general surgeons, nearly half of GPs, 40% of urologists, and approximately one-third of gynecologists were able to correctly diagnose cutaneous lesions localized in the axillary region at Hurley III stage. These diagnostic rates, while indicative of some degree of recognition of HS as a distinct clinical entity, remain suboptimal. The misdiagnosis results in directing the patient to an inappropriate physician, prolonging the time to establish a correct diagnosis and introducing appropriate treatment. Meriting particular attention is the fact that the prolonged diagnostic pathway fosters a sense of resignation among HS patients, potentially leading to hesitation regarding the necessity of subsequent appointments, thereby further prolonging diagnostic delays and diminishing patients’ quality of life.

In the case of the genital area of a male patient, pertaining Hurley II stage, the most frequently chosen diagnosis among non-dermatologist physicians was furunculosis. Considering that furunculosis is an infectious disease, healthcare providers might advocate for enhanced hygiene measures among their patients. It may engender feelings of stigma among patients, thereby precipitating a breakdown in the trust dynamic between patient and provider. The second most frequently proposed diagnosis among those clinicians was lymphogranuloma venereum and noduloulcerative syphilis. Given that lymphogranuloma venereum and syphilis are sexually transmitted infections (STIs), erroneously labeling a patient with these conditions could not only result in inappropriate treatment but also precipitate profound psychosocial consequences [11].

The diagnosis of HS in the female patient displaying Hurley I lesions located in the intimate area was established in 29.92% of cases. This suggests that subtle early-stage presentations, particularly in anatomically sensitive areas, may contribute to diagnostic hesitation or uncertainty. Additionally, gender-specific symptom expression could further complicate clinical assessment. Among dermatologists, only 58.45% provided a proper diagnosis. It is noteworthy that 69.16% of clinicians opted for referral to a dermatologist. It is notable that even among those referred, just over half would receive a correct diagnosis and, consequently, may have chances for appropriate treatment.

In the context of Hurley III lesions located on the buttocks, the most frequently indicated diagnoses were HS, followed closely by pilonidal cyst and the cutaneous manifestation of Crohn’s disease. The diagnosis of a pilonidal cyst frequently prompted the referral of this patient to a general surgeon. Abscess incision, while offering swift and effective pain relief, does not alter the long-term course of HS, as inflammatory skin alterations promptly recur [1]. Furthermore, the course of this disease evolves from initial inflammatory manifestations to tissue destruction [8]. The diagnosis of cutaneous manifestation of Crohn’s disease, however, necessitated referral to a gastroenterologist as the primary physician overseeing the patient’s care. Crohn’s disease typically requires management with immunosuppressive medications and dietary modifications, while HS treatment often involves a combination of antibiotics, anti-inflammatory drugs, and surgical interventions [12]. Thus, accurate diagnosis is crucial to initiate timely and tailored interventions, optimize patient outcomes, and mitigate the risk of disease progression or complications in HS.

The lack of familiarity with this condition may constitute the underlying cause behind these inaccurately rendered diagnoses. The data suggest that HS is not adequately covered in medical school curricula, as in a study conducted by Alhawsawi et al. [13] only 21.2% of the participants cited their medical education as a source of their knowledge about HS. The majority of the knowledge seems to be acquired through clinical practice and informal sources rather than structured educational settings. This indicates a gap in formal medical education regarding HS, necessitating enhanced educational focus on this condition during medical training. This educational gap contributes to the misclassification of the disease and subsequent delays in diagnosis [5,6]. Indeed, in the survey study conducted by Lopes et al. [6], which aimed to evaluate the awareness of HS among GPs, surveyed clinicians reported encountering numerous patients with this condition each month. However, there appears to be a deficiency in understanding disease correlations and, notably, treatment approaches. Furthermore, it was noted that patients are not consistently referred appropriately, with many cases not being referred to dermatologists. A study by Esme P. et al. [5] evaluated the understanding of HS among GPs, revealing that only 23.7% felt confident in diagnosing the condition. Furthermore, approximately 63% mistakenly identified HS as an infectious disease, contradicting its actual pathophysiology. Our findings support these observations and additionally expand on them by including a wider range of medical specialties and a larger group of participants, providing a broader picture of the educational shortcomings in HS recognition.

Moreover, Stormo et al. [14] conducted research revealing that obstetrician–gynecologists delivered well-woman preventive care to 44% of women, with women under 50 years demonstrating a greater propensity to seek such care from obstetrician–gynecologists rather than primary care physicians. Consequently, obstetrician–gynecologists possess a distinct opportunity to identify HS at an early stage since many women attend annual examinations, and the groin, axilla, and inframammary regions—frequently affected by this condition—are areas routinely assessed during these visits. Diagnosing HS may pose challenges for gynecologists since the symptoms can mimic other conditions such as Bartholin cysts, lymphogranuloma venerum, granuloma inguinale or steatocystoma multiplex [15]. These difficulties may be exacerbated by the fact that female patients, compared to male counterparts, exhibit symptoms of lesser disease severity, which, according to the findings of our study, poses greater recognition obstacles [16].

Medical journals serve as pivotal resources for enhancing the understanding and management of HS among diverse clinicians. Upon reviewing the literature, there is a notable deficiency in the literature available within medical journals focused on specialties such as obstetrics and gynecology (OBGYN) and family medicine, which serve as primary healthcare providers for HS patients [17] It underscores a substantial gap in the literature available to frontline healthcare providers who frequently encounter and manage HS cases in their clinical practice. Furthermore, in the context of textbooks utilized for final specialty examinations in Poland, it is noted that for general practitioners [18], as well as in gynecology and obstetrics [19], general surgery [20], and urology [21], there is a notable absence of any information pertaining to HS. This omission highlights a significant gap in the educational resources provided to medical students and specialists in these disciplines, indicating a need for curriculum updates to include comprehensive coverage of this complex condition.

The increasing academic interest and awareness of HS is evident from an analysis of scholarly publications indexed in PubMed. Between 2007 and 2015, there were 758 papers published on this topic, which significantly rose to 3,624 publications from 2016 to 2023 (the search string included “hidradenitis suppurativa” OR “acne inversa” OR “Verneuil’s disease” OR “apocrine acne”). The growing body of research and enhanced awareness are positive developments, yet they only begin to scratch the surface of the challenges posed by HS. In terms of dermatologists’ education regarding HS, significant attention has been devoted to this disease entity in Poland over the last decade. In 2019, Poland hosted the 8th European Hidradenitis Suppurativa Foundation (EHSF) Conference. Additionally, annual gatherings specifically dedicated to HS, known as “HS Day”, are held to foster dialogue and share advancements in the management and understanding of this condition. Moreover, at the European Academy of Dermatology and Venereology (EADV) conferences, subspecialty sessions dedicated to HS have been conducted. It is of substantial relevance that the content primarily pertains to therapeutic innovations, new epidemiological data, pathophysiological insights, and socio-economic considerations. It is conceivable that there may be a dearth of lectures elucidating the diversity of clinical presentations, including manifestations in earlier stages of severity, which are pivotal for accurate diagnosis.

Beyond specialty-related differences, the impact of training stages on diagnostic accuracy was mostly evident within dermatology. In this group, more recent training was associated with higher recognition rates, with performance declining among more experienced physicians. In other specialties, diagnostic accuracy remained uniformly low regardless of career stage—with the exception of GPs who showed a significant drop in performance in the severe case (Hurley III, buttocks lesions). These findings suggest that clinical experience alone does not necessarily improve HS recognition and highlight the need for ongoing education across all career stages.

Concerning the limitations of the study, one limitation is the potential for selection bias due to the method of data collection. Since the survey was conducted at scientific conferences, only physicians attending these events had the opportunity to participate. This could result in a sample that is not fully representative of all physicians across various specialties, potentially leading to skewed results. Furthermore, the surveys were developed by experts of HS, distributed, and collected by dermatologists, but they were not validated. Be that as it may, the presence of dermatologists at the conferences provided an avenue for respondents to seek clarification or additional information, potentially enhancing the accuracy of responses.

To address the challenges identified in our study, future efforts should focus on implementing practical, evidence-based strategies to improve HS recognition among non-dermatologist physicians. Our data indicate particularly low diagnostic accuracy in gynecology, urology, and family medicine across all training stages, underlining the need for targeted interventions. Given that many clinicians encounter HS without prior formal training, continuing medical education (CME) formats tailored to time-limited professionals are particularly important. Structured e-learning platforms, interactive case-based modules, and brief decision-making workshops can offer accessible learning opportunities. Moreover, innovative microlearning approaches have shown remarkable potential; for example, a WhatsApp-based educational campaign in Argentina significantly improved GPs’ understanding of HS pathophysiology and management, with these gains sustained at a nine-month follow-up [22]. Such low-cost, high-impact interventions could be easily adapted in other healthcare settings.

Importantly, expanding HS awareness beyond dermatology remains crucial. Clinicians from specialties such as gynecology, general surgery, urology, and family medicine are often the first point of contact for patients, yet HS is under-represented in their core training and professional resources. Given the observed misclassification patterns in these fields—such as syphilis, lymphogranuloma venereum, and pilonidal disease—targeted education should emphasize differential diagnosis in HS-prone anatomical areas. Educational outreach—including HS-focused presentations at specialty conferences, dedicated sessions on recurrent boils and abscess differentials, and publications in non-dermatological journals—may increase diagnostic accuracy in these fields. Efforts should also include the development and dissemination of referral algorithms or checklists tailored to each specialty, helping guide appropriate management and specialist consultation.

Notably, even among dermatologists, the recognition of early-stage HS was suboptimal, especially in cases involving mild lesions. This was evident in Hurley I and II scenarios, where dermatologists—although outperforming other fields—did not achieve full recognition accuracy. Although HS is now widely discussed within the dermatology community, our findings highlight the ongoing need to strengthen diagnostic skills, suggesting that educational gaps persist even within dermatology, particularly in relation to early presentations. To improve diagnostic accuracy, dermatology training should place greater emphasis on the clinical variability of HS. Incorporating high-quality clinical images, structured diagnostic algorithms, and interactive case-based teaching sessions into residency programs may enhance early recognition. In addition, expanding access to diverse clinical cases through rotations in academic centers with a higher number of HS patients, or through curated digital case libraries, could help trainees and practicing dermatologists develop greater diagnostic confidence at earlier disease stages.

Facilitating collaboration between dermatologists and other specialists—for example, through shared referral pathways, interdisciplinary consultations, or joint case discussions—may support both knowledge exchange and more coordinated care. In this context, the integration of multidisciplinary teams (MDTs), with dermatologists and surgeons forming the core, may be particularly beneficial for optimizing the diagnostic and therapeutic pathway in HS.

Altogether, these strategies—ranging from mobile-based micro-education to formal cross-specialty collaboration—represent realistic and scalable ways to reduce the diagnostic delay and fragmentation of care experienced by patients with HS. Their implementation could support earlier recognition, more accurate referrals, and ultimately, more timely and effective treatment for this often overlooked and burdensome disease.

## 5. Conclusions

HS constitutes a clinical entity characterized by multifaceted diagnostic challenges across diverse medical specialties, even dermatologists. Visiting multiple healthcare providers often results in various misdiagnoses for patients, causing frustration, elevated expenses, and sometimes leading to improper treatments. Targeted educational interventions should aim to bridge the gaps of knowledge of HS, striving for earlier and more accurate HS diagnoses to facilitate timely and effective therapeutic interventions, thereby improving patient outcomes.

## Figures and Tables

**Table 1:** The distribution of diagnoses made by all physicians for the Hurley III case in Figure 1. The table includes the names of diseases along with the percentage of respondents selecting each diagnosis.

Disease	Percentage [%] of Answers
hidradenitis suppurativa	58.02
furunculosis	15.27
colliquative tuberculosis	7.33
syphilis	5.8
metastases to the axillary lymph nodes	5.64
ulceration	2.74
actinomycosis	1.83
keloids	0.92
folliculitis	0.46
panniculitis	0.31
tinea corporis	0.31
necrotizing fasciitis	0.31
tumor	0.31
cat scratch disease	0.15
condylomata acuminata	0.15
acne conglobata	0.15
ecthyma	0.15
plague	0.15

**Table 2:** HS (Hurley III, open-ended question) diagnostic accuracy (%) by specialty and career stage. Between-group comparisons were conducted using chi-square and Fisher–Freeman–Halton exact tests, depending on data distribution.

Specialty	Residents (%)	<5 Years (%)	5–10 Years (%)	>10 Years (%)	*p*-Value (Across Career Stages)
Dermatologists	100	100	92.31	92.45	NS
GPs	62.26	52.63	29.41	31.03	*p* < 0.05
Urologists	40.85	16.67	50.00	60.00	NS
Gynecologists	29.17	33.33	28.57	31.71	NS
Surgeons	59.68	75.00	72.22	55.26	NS
*p*-value (across specialties)	*p* < 0.001	*p* < 0.001	*p* < 0.001	*p* < 0.001	

NS—not significant.

**Table 3:** HS (Hurley II, close-ended question) diagnostic accuracy (%) by specialty and career stage. Between-group comparisons were conducted using chi-square and Fisher–Freeman–Halton exact tests, depending on data distribution.

Specialty	Residents (%)	<5 Years (%)	5–10 Years (%)	>10 Years (%)	*p*-Value (Across Career Stages)
Dermatologists	90.57	100.00	69.23	66.04	*p* < 0.05
GPs	24.53	26.32	5.88	6.90	NS
Urologists	18.31	22.22	50.00	20.00	NS
Gynecologists	16.67	21.21	0.00	14.63	NS
Surgeons	17.74	36.11	44.44	26.32	NS
*p*-value (across specialties)	*p* = 0.001	*p* = 0.001	*p* = 0.007	*p* < 0.001	

NS—not significant.

**Table 4:** HS (Hurley I, close-ended question) diagnostic accuracy (%) by specialty and career stage. Between-group comparisons were conducted using chi-square and Fisher–Freeman–Halton exact tests, depending on data distribution.

Specialty	Residents (%)	<5 Years (%)	5–10 Years (%)	>10 Years (%)	*p*-Value (Across Career Stages)
Dermatologists	60.38	82.61	53.85	48.08	NS
GPs	13.21	13.16	5.88	4.17	NS
Urologists	12.68	0	33.33	7.14	NS
Gynecologists	16,67	12.12	0	8.98	NS
Surgeons	12.90	27.78	44.44	18.42	NS
*p*-value (across specialties)	*p* = 0.001	*p* = 0.001	*p* = 0.017	*p* = 0.001	

NS—not significant.

**Table 5:** HS (Hurley III, close-ended question) diagnostic accuracy (%) by specialty and career stage. Between-group comparisons were conducted using chi-square and Fisher–Freeman–Halton exact tests, depending on data distribution.

Specialty	Residents (%)	<5 Years (%)	5–10 Years (%)	>10 Years (%)	*p*-Value (Across Career Stages)
Dermatologists	90.57	95.65	69.23	56.60	*p* < 0.05
GPs	22.64	13.16	23.53	0	*p* < 0.05
Urologists	12.68	11.11	33.33	0	NS
Gynecologists	20.83	15.15	28.57	9.76	NS
Surgeons	9.68	30.56	44.44	23.68	NS
*p*-value (across specialties)	*p* < 0.001	*p* < 0.001	NS	*p* < 0.001	

NS—not significant.

## Data Availability

The data underlying this article will be shared on reasonable request to the corresponding author.

## Abbreviations

The following abbreviations are used in this manuscript:

HS	Hidradenitis Suppurativa;
GPs	General Practitioners;
ICD-10	International Classification of Diseases, Tenth Revision;
STIs	Sexually Transmitted Infections;
OBGYN	Obstetrics and Gynecology;
EHSF	European Hidradenitis Suppurativa Foundation;
EADV	European Academy of Dermatology and Venereology.

## Appendix A

A survey distributed among physicians of various specializations, paper version.

**Age (in years)
**

**……………………………………
**

**Gender**

o Female

o Male

**Type of completed or ongoing specialty training
**

o Family Medicine/Internal Medicine

o Dermatology

o Surgery

o Gynecology and Obstetrics

o Urology

**Stage of medical specialization
**

o Resident Physician

o Specialist Physician within 5 years of completing specialty training

o Specialist Physician 5–10 years post-specialty training

o Specialist Physician more than 10 years post-specialty training

1. **What diagnosis should be made for the patient shown in the image below? [the image shows the armpit area]**

The patient reports recurrent symptoms for approximately six months.

(This is the only open-ended question in the survey.)

Your response:

………………………………………………………………………………………………

2. **What disease entity is presented in the image below? [the image shows the genital area]**

The patient reports recurrent symptoms for approximately six months.

*Lymphogranuloma venereum*

*Penis carcinoma metastases*

*Acne conglobata*

*Furunculosis*

*Scrofuloderma*

*Hidradenitis suppurativa*

*Cutaneous Crohn’s disease*

*Lymphadenopathy*

*Cat scratch disease*

*Nodulo-ulcerative syphilis*

*None of the above*

**To which specialist should the patient from the previous image be referred?
**

o Infectious Disease Physician

o Surgeon

o Gastroenterologist

o Urologist

o Dermatologist

o Oncologist

o Family Medicine Physician/General Practitioner

3. **What disease entity is presented in the image below? [the image shows the anogenital area]**

The patient reports recurrent symptoms for approximately six months.

*Acne conglobata*

*Furunculosis*

*Batholinitis*

*Hidradenitis suppurativa*

*Cutaneous Crohn’s disease*

*Scrofuloderma*

*Lymphogranuloma venereum*

*Lymphadenopathy*

*Vulvar carcinoma metastases*

*Granuloma inguinale*

Cat *scratch disease*

*Nodulo-ulcerative syphilis*

*Steatocystoma multiplex*

*None of the above*

**To which specialist should the patient from the previous image be referred?
**

o Oncologist

o Surgeon

o Infectious Disease Physician

o Gastroenterologist

o Family Medicine Physician/General Practitioner

o Dermatologist

o Gynecologist

o Urologist

4. **What disease entity is presented in the image below? [the image shows the buttock area]**

The patient reports recurrent symptoms for approximately six months.

*Pilonidal* *cyst*

*Acne conglobata*

*Cutaneous Crohn’s disease*

*Hidradenitis suppurativa*

*Scrofuloderma*

*Lymphadenopathy*

*Nodulo-ulcerative syphilis*

*Furunculosis*

*Cat scratch disease*

*None of the above*

**To which specialist should the patient from the above image be referred first?
**

o Gastroenterologist

o Family Medicine Physician/General Practitioner

o Surgeon

o Dermatologist

o Oncologist

o Urologist

o Infectious Disease Physician
